# Pulmonary resident memory T cells in respiratory virus infection and their inspiration on therapeutic strategies

**DOI:** 10.3389/fimmu.2022.943331

**Published:** 2022-08-12

**Authors:** Meng Zhang, Na Li, Yanchao He, Tianyun Shi, Zhijun Jie

**Affiliations:** ^1^ Department of Pulmonary and Critical Care Medicine, Shanghai Fifth People’s Hospital, Fudan University, Shanghai, China; ^2^ Center of Community-Based Health Research, Fudan University, Shanghai, China

**Keywords:** immune memory, tissue-resident memory T cells, respiratory syncytial virus, influenza, SARS-CoV-2, therapeutic strategies

## Abstract

The immune system generates memory cells on infection with a virus for the first time. These memory cells play an essential role in protection against reinfection. Tissue-resident memory T (TRM) cells can be generated *in situ* once attacked by pathogens. TRM cells dominate the defense mechanism during early stages of reinfection and have gradually become one of the most popular focuses in recent years. Here, we mainly reviewed the development and regulation of various TRM cell signaling pathways in the respiratory tract. Moreover, we explored the protective roles of TRM cells in immune response against various respiratory viruses, such as Respiratory Syncytial Virus (RSV) and influenza. The complex roles of TRM cells against SARS-CoV-2 infection are also discussed. Current evidence supports the therapeutic strategies targeting TRM cells, providing more possibilities for treatment. Rational utilization of TRM cells for therapeutics is vital for defense against respiratory viruses.

## Introduction

**Figure 1:**
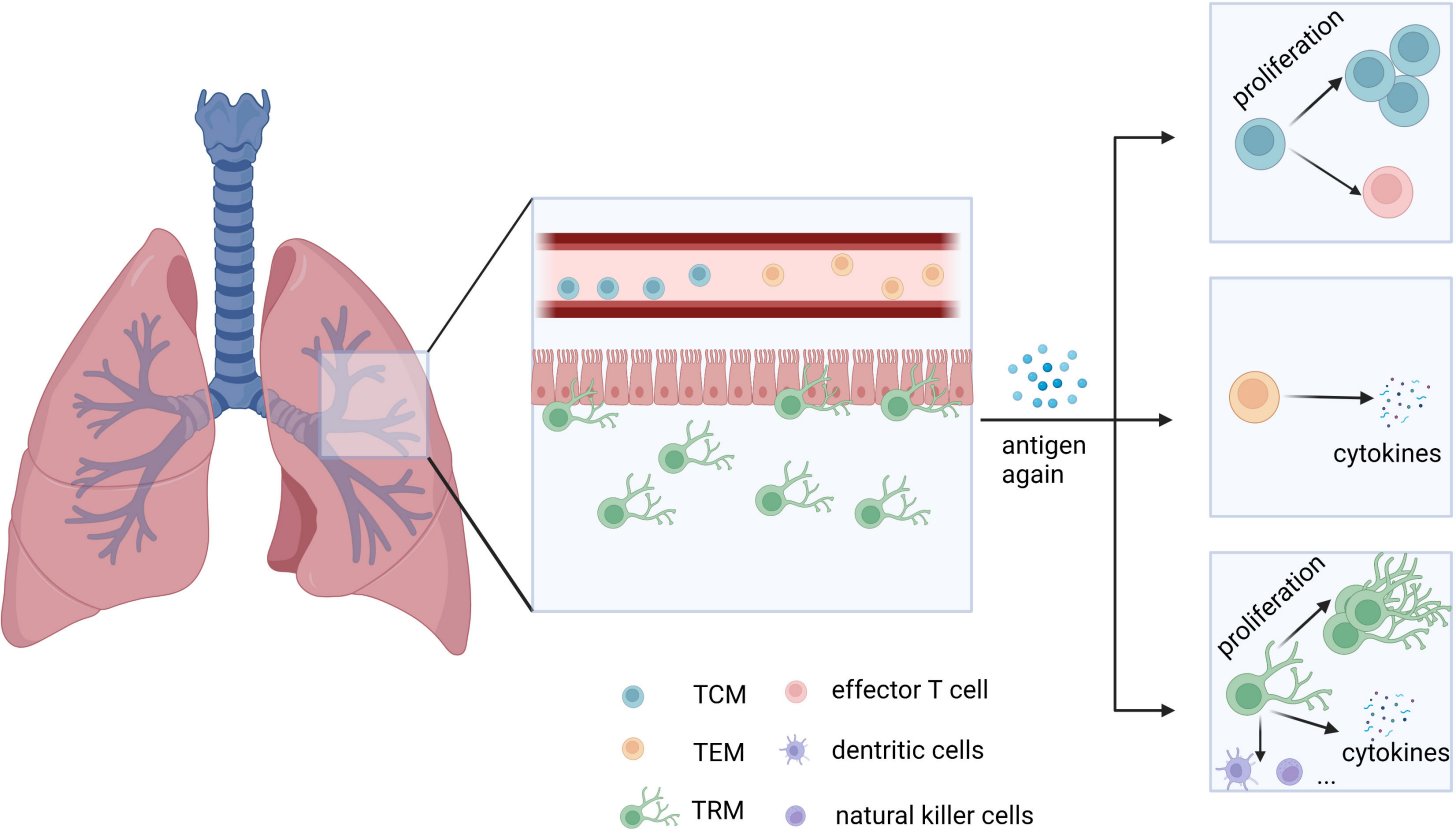
General distribution of TEM, TCM, and TRM cells in the lungs and their main mode of function. TEM and TCM cells migrate in circulation, while TRM cells reside in tissues. When stimulated by antigens again, TCM cells rapidly proliferate and differentiate, while TEM cells secrete effector molecules like granzyme B, similar to CD8+ T cells. TRM cells exert a rapid and critical immune response on local tissues. TCM: central memory T cells. TEM: effector memory T cells. TRM: tissue-resident memory T cells.

Being exposed to various stimuli, the host forms an immune defense to protect the body for the first viral encounter and preserve immunological memory to prepare for reinfection. Memory T cells are the backbone of this process and are classified as central memory T (TCM) cells and effector memory T (TEM) cells (1). Generally, TCM cells express chemokine receptor CCR7, homing molecule CD62L, and Krupple-Like Factor 2 (KLF2), circulating in the peripheral lymph system for surveillance of secondary lymphoid organs and recalling of proliferative responses. Distribution of TCM cells includes peripheral tissues, immune organs, and lymph nodes (1–3). TEM cells are mainly located in non-lymphocyte tissues and organs, with the expression of CCR7 and CD62L being hardly detected. TEM cells participate in the body circulation and migrate to the peripheral inflammatory tissues to induce rapid effects (1–3). Later, resident and self-sustaining memory T cells surviving in the non-lymphoid tissues, namely TRM cells, were identified by parabiosis and tissue transplantation experiments (4, 5). Despite the shared cell marker CD44^+^ of memory T cells, TRM cells highly express CD69 and/or CD103 instead of CCR7 and CD62L (6, 7). They are the most abundant subset of memory T cells and reside in the non-lymphoid tissues for a long time, contributing to rapid and critical protective immune responses (8). In addition to proliferation, TRM cells secrete cytokines and pass the signals to other cells like natural killer cells to dominate local memory responses (9, 10) (**Table 1**; **Figure 1**).

**Table 1:** basic traits of effector memory T cells, central memory T cells and resident memory T cells.

		TEM	TCM	TRM
**Location**		peripheral tissues, immune organs and lymph nodes.	mainly non-lymphoid tissues and organs	non-lymphoid tissues, including lung, intestine, brain,
**Function**		stimulated by antigen again, it can rapidly proliferate and differentiate	participate in the circulation of the body, and can migrate to peripheral inflammatory tissues to induce rapid effects	resident in a particular tissue, exerting a rapid and critical protective immune response in local tissues
**Surface markers**	**shared**	CD44+		
	**distinguished**	CD62L^+^, CCR7^+^, KLF2^+^, CD69^-^, CD103-	CD62L^-^, CCR7^-^, KLF2^+^, CD69^-^, CD103^-^	CD62L^-^, CCR7^-^, KLF2^-^, CD69^+^, CD103^+^

TRM cells found in both the upper and lower respiratory tract play an important role in the localized defense against respiratory infections. Due to rapid spread through air, respiratory viruses pose a considerable threat to humans. In recent years, respiratory viruses have stood on the stage of global pandemic. Having a deeper understanding of the role of immune cells in protecting the respiratory tract helps curb the spread of respiratory disease, casting light on new strategies. In this review, we summarize the characteristics of TRM cells and their complicated roles during RSV, Influenza, and SARS-CoV-2 infection. Several potential preventive and therapeutic strategies based on TRM cells are discussed.

## Development of TRM cells in respiratory Tract

Once viruses attack the respiratory tract, DNGR1^+^ dendritic cells (DCs) preferentially migrate to the mediastinal lymph nodes (medLNs). These DCs activate naïve T cells to differentiate into precursor effector T cells (CD8^+^ T cells), some of which further migrate to the nasal cavity or lungs and convert into TRM cells (2, 11). Precursor CD4^+^ T cells can also transform into tissue-resident memory T cells. Generally, the phenotype of pulmonary CD4^+^ TRM cells is regarded as CD69^+^CD103^-^, while the phenotype of CD8^+^ TRM cells is CD69^+^CD103^+^ (2). Other phenotypes of TRM cells in respiratory viruses are summarized in **Table 2**. Multiple signaling pathways contribute to the initiation and maintenance of TRM cells.

**Table 2:** phenotypes of CD8^+^ and CD4^+^ TRM cells in respiratory viruses, as well as cytokines and transcription factors in the regulation of pulmonary TRM cells.

	CD8^+^ TRM	CD4^+^ TRM
**Phenotype**		
RSV	Human CD69^+^CD103^+^ Mouse CD69^+^CD103^+^	Human CD69^+^CD103^+/-^ Mouse CD69^+^CD103^-^(CD49d^+^CD11a^hi^)
Influenza	Human CD69^+/-^CD103^+/-^(HLA-DR^+^, NKG2A^+^, CD11a^+^, CD49a^+^, CD101^+^, PD-1^hi/lo^) Mouse CD69^+/-^CD103^+/-^(IFITM3^+^, PD-1^hi^, CCR7^-^, CD11a^+^, CD49a^+^, Ly6C^-^)	Human CD69^+^CD103^+/-^ (PD-1^hi^, CD49d^+^, CD101^+^) Mouse CD69+(CD11a^+^, PD-1^+^, FR4^lo/hi^)
SARS-CoV-2	Human CD69^+^CD103^+/-^(HLA-DR^+^, PD-1^+^, PSGL-1^+^) Mouse CD69^+^CD103^+/-^	Human CD69^+^CD103^+/-^(HLA-DR^+^, PD-1^+^) Mouse CD69+
**References**	(9, 10) (11–26) (27–29)	(30, 31) (11, 17, 32, 33) (27–29)
**Development**		
Generation	(+) TNF-α, IL-33, TGF-β, IL-15, Blimp1, Runx3, Bhlhe4, Notch (-) KLF2, Tcf1, T-bet, Eomes	(+) IL-15, IL-2, Notch (-) T-bet, Eomes
Maintenance	(+) Blimp1, Runx3, Bhlhe4, Notch, CD69 (-) KLF2, T-bet, Eomes, S1PR1, CCR7	(+) IL-7, Notch, CD69 (-) S1PR1
**References**	(34–44) (36, 37, 39, 43, 45–49)	(45, 50–52) (34, 45, 49, 53)

### Initiation and generation of TRM cells

The initiation and generation of TRM cells involves different signaling pathways and transcriptional factors that vary in different tissues. Local antigen stimulation triggers the initiation of TRM cells in various tissues, including the lungs, but not in the nasal cavity (36). Cytokines like TGF-β, interleukin (IL), and TNF-α are also involved in the regulation of TRM cell development. TGF-β has multiple effects in adaptive immunity with complex biological activities (37). Pulmonary CD103 expression by CD8^+^ TRM cells relies on TGF-β, which is regulated by CD1c^+^ DCs (38). TGF-β, IL-33, and TNF-α can also inhibit Krupple-Like Factor 2 (KLF2) expression to downregulate the expression of sphingosine-1-phosphate receptor (S1PR1), resulting in elevated CD69 expression in CD8^+^ TRM cells (39). Furthermore, TGF-β downregulates other transcription factors in the lungs to promote the establishment of pulmonary CD8^+^ TRM cells, such as T-bet, eomesodermin (Eomes), and transcription factor 1 (40, 41). In mouse experiments, high expression levels of T-bet, Eomes, and transcription factor 1 inhibit CD103 expression (41–43). Expression of CD103 can be stimulated either directly by Runx3 or Blimp1, which has been proven in the development of gut TRM cells (44, 50). Since TGF-β is upstream of Runx3 and Blimp1 in the gut, it might also regulate CD103 in the lungs by the same pathway. Additionally, transcription factor Bhlhe4 can also increase the expression of CD103 directly or indirectly *via* the recruitment of Runx3 (42, 51).

IL-15 reduces T-bet expression to mediate CD8^+^ TRM cell generation together with TGF-β (40). IL-33 and IL-12 increase Blimp-1 while inhibiting Eomes and TCF-1, promoting the generation of pulmonary CD8^+^ TRM cells (45). Additionally, IL-15 and IL-2 aid in the formation of CD4^+^ TRM cells in asthmatic mouse models (52, 54). In addition to ILs, other regulators shared by CD4^+^ and CD8^+^ T cells include low levels of transcription factors T-bet and Eomes and upregulated Notch signaling (40, 42, 55, 56). However, the detailed signaling mechanisms of CD4^+^ TRM cell generation are still unclear and more relevant studies are necessary.

Cell environment *in situ* can also influence TRM cell formation. Lung macrophages can regulate TRM cell formation, while their effects according to different experiments seem inconsistent. In influenza mouse models, macrophages exert negative effects (46), while human pulmonary macrophages provide costimulatory signals during CD8^+^ TRM cells generation (21). CD4^+^ T cells and T regulatory cells expressing T-bet assist in the formation of CD103^+^ pulmonary CD8^+^ TRM cells (29, 47).

### Maintenance of TRM cells

The distinguishing characteristic of TRM cells is that they can sustain in non-lymphoid tissue for a long time, acting as the assault force in secondary infection. Repair-associated memory depots are the major niche where CD8^+^ TRM cells are stored in the lungs (48). Transcription factors play vital roles in the survival of TRM cells. Runx3 and Blimp1, which inhibit transcription factor 1, maintain the survival of CD8^+^ TRM cells by suppressing egress receptors CCR7 and S1PR1 (49, 57). S1PR1 induces the chemotaxis of sphingosinol-1-phosphate, mediating the expulsion of T cells from the tissues (53). Therefore, FTY720, an S1PR1 agonist, is often used in research focused on TRM cells to inhibit peripheral lymphatic circulation (58). KLF2, which promotes S1PR1 expression, is downregulated after CD8^+^ TRM cells enter non-lymphoid tissues (39). Furthermore, CD8^+^ TRM cells are sustained in a functional epigenetic state by Bhlhe4 (51). Reduction of T-bet and Eomes also enhances the maintenance of CD8^+^ TRM cells (40).

Additionally, some transcription factors regulating maintenance are common to both CD4^+^ and CD8^+^ TRM cells. Notch promotes the survival of CD4^+^ and CD8^+^ TRM cells (42, 55). CD69 antagonizes S1PR1 and inhibits TRM cells excretion (59), and research demonstrates that TRM cells cannot sustain in CD69-/- mice (27). With reference to CD4^+^ TRM cells, IL-7 is critical for the maintenance of CD4^+^ TRM cells in the lungs (60). Distinct from CD8^+^ TRM cells, inducible bronchus associated lymphoid tissue is the major niche for pulmonary CD4^+^ TRM cells (61).

**Figure 2:**
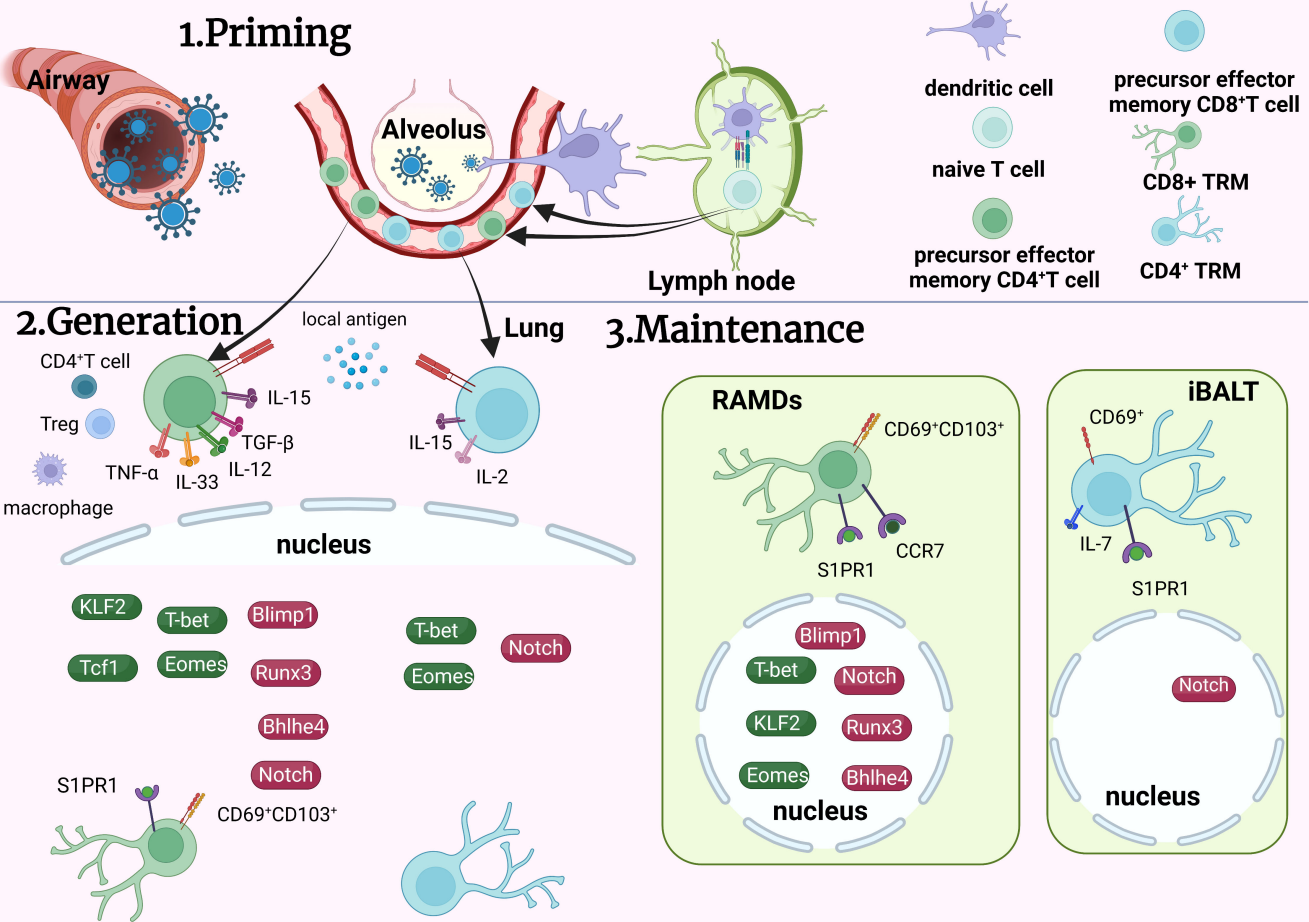
Development of TRM cells in the lung. On contact with the virus, specific dendritic cells migrate to lymph nodes to communicate with naïve T cells and activate the generation of effector T cells. Some effector T cells migrate to the lungs and transform into TRM cells by various signaling pathways. CD8^+^ TRM cells accumulate in RAMDs and CD4^+^ TRM cells accumulate in iBALs after formation. To maintain TRM cells in the lungs, many transcription factors coordinate with each other to achieve overall functionality. TRM, tissue resident memory T cells; RAMDs, repair associated memory depot; iBALs, inducible bronchus associated lymphoid tissue; APC, antigen presenting cells; Green icons, inhibitory roles; red icons, facilitating roles.

**Figure 3:**
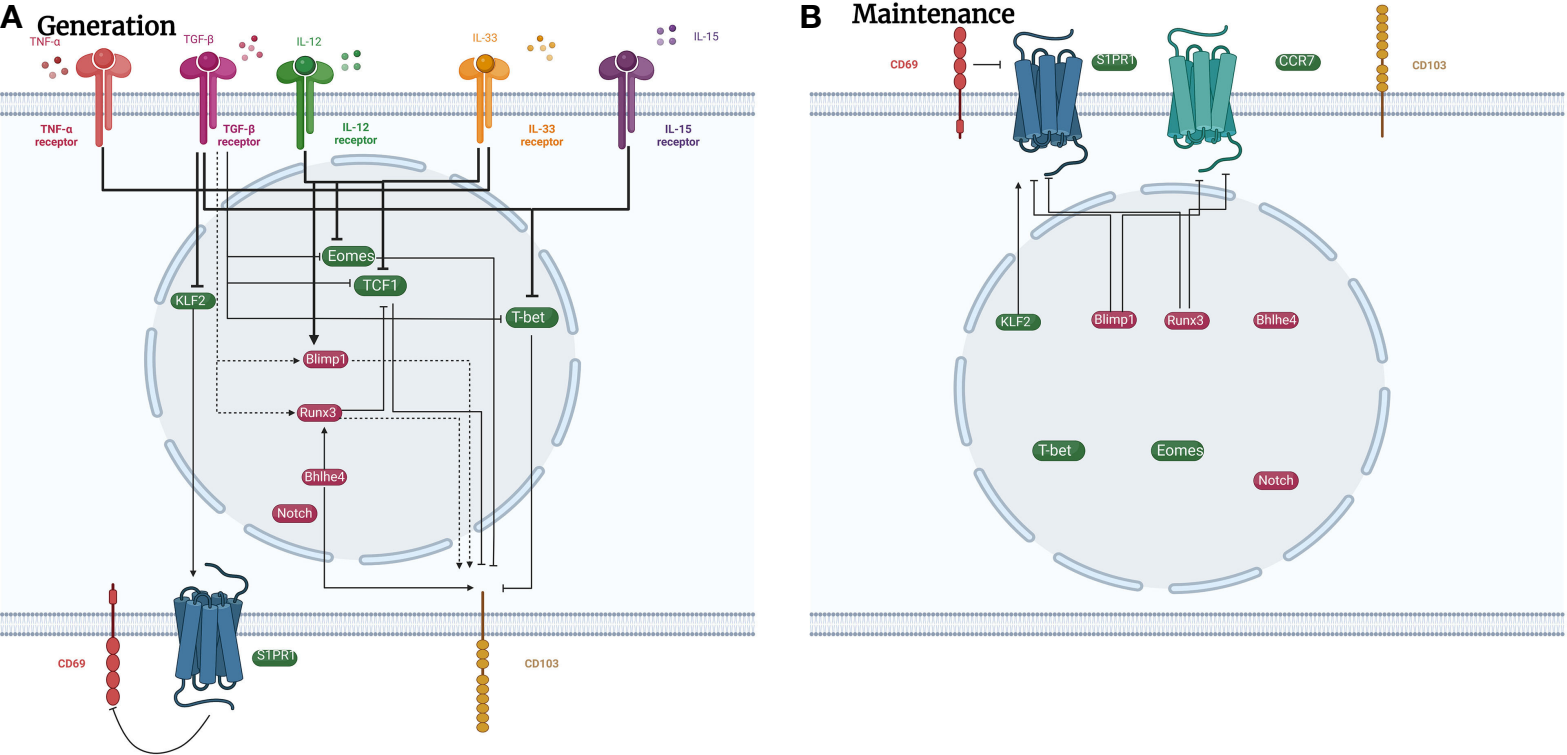
Signaling pathways in regulating the generation and maintenance of pulmonary CD8^+^ TRM cells. Plenty of signaling pathways regulate the development of CD8^+^ TRM cells. **(A)** TNF-α, IL-33, TGF-β, and IL-15 promote the development of CD8^+^ TRM cells by increasing the expression of transcription factors such as Runx3, Blimp1, and Bhlhe4, and decreasing the expression of transcription factors such as Eomes, T-bet, and KLF2. **(B)** Transcription factors such as Blimp1 and Runx3 inhibit the expression of cell surface receptors CCR7 and S1PR1 to maintain the presence of CD8^+^ TRM cells in the lungs. Cell surface marker CD69 can also decrease the expression of S1PR1 to sustain CD8^+^ TRM cells. green icons: inhibitory roles; red icons: facilitating roles. dotted line: possible impact.

Evidently, generation, maintenance, and regulation of TRM cells in the lungs is complicated (**Figure 2** **;** **Figure 3** **;** **Table 2**) and involves a variety of factors. Additional details are needed regarding the mechanism of TRM cell regulation to understand their traits. Thereafter, we can control the physiological process managed by them to regulate immunological homeostasis.

## Basic functions and ‘Workflow’ of TRM cells

As fundamental components of the immune network, TRM cells monitor local homeostasis (8). Furthermore, they participate in the response to external stimuli and internal abnormalities, maintaining homeostasis (13, 62). TRM cells patrol colonization site, recognize reinfection antigen or tumor-associated antigens, secrete pro-inflammatory cytokines, and release cytotoxic particles to eliminate these antigens (63). Contrastingly, signals induced by TRM cells break tissue homeostasis with hybrid populations of white blood cells. For instance, independent of circulating immune memory cells, CD4+ TRM cells are considered the primary reactive immune memory cells in the early period of asthma in house dust mite asthmatic mouse model (54). Clearly, functions of TRM cells are diverse under different circumstances.

TRM cells work mainly in two steps: the first step is scanning. Long resident memory T cells slowly and randomly migrate to previously infected tissues. In the inflammatory tissue, T cells increase their movement five-fold in the mucosa and bind to fibronectin with integrins CD103 and CD49a, which are important markers of TRM cells in the lungs (64, 65). Local immune surveillance against relapse or reinfection is enhanced by TRM cells scanning the environment (66). On contact with the antigen, TRM cells initiate the second step: clearance. In addition to the direct control of the pathogen, some tissue-specific CD8^+^ TRM cells sustain constitutive granzyme B expression, mediate extracellular toxicity (67, 68), and eliminate pathogens by non-cytolytic progress (69). Furthermore, they convey this information to neighboring cells, stimulating localized memory responses. TRM cell-dependent parenchymal immunity, including innate and adaptive immune activation, triggers the induction of locally protective antiviral status (9, 10).

Overall, TRM cells can settle in a particular niche for a long time, leading to a rapid and critical protective immune response in the local tissues. Through the two steps —scan and clearance — TRM cells are involved in infection protection, tumor control, and monitoring homeostasis to play various roles in different diseases.

## Role of TRM cells in respiratory virus infection

### Respiratory syncytial virus

Since it was first isolated in Chimpanzee with respiratory illness in 1957 (70), RSV has evolved as a vast threat to children, adults, and the elderly, especially infants. RSV-associated hospitalization rates are highest in infants younger than six months and almost all children get infected with RSV by the age of two (71). A large systematic study has shown that RSV was responsible for 2.0% and 3.6% of deaths in children aged 0–60 months and 28 days–6 months, respectively, in 2019 (72).

**Figure 4:**
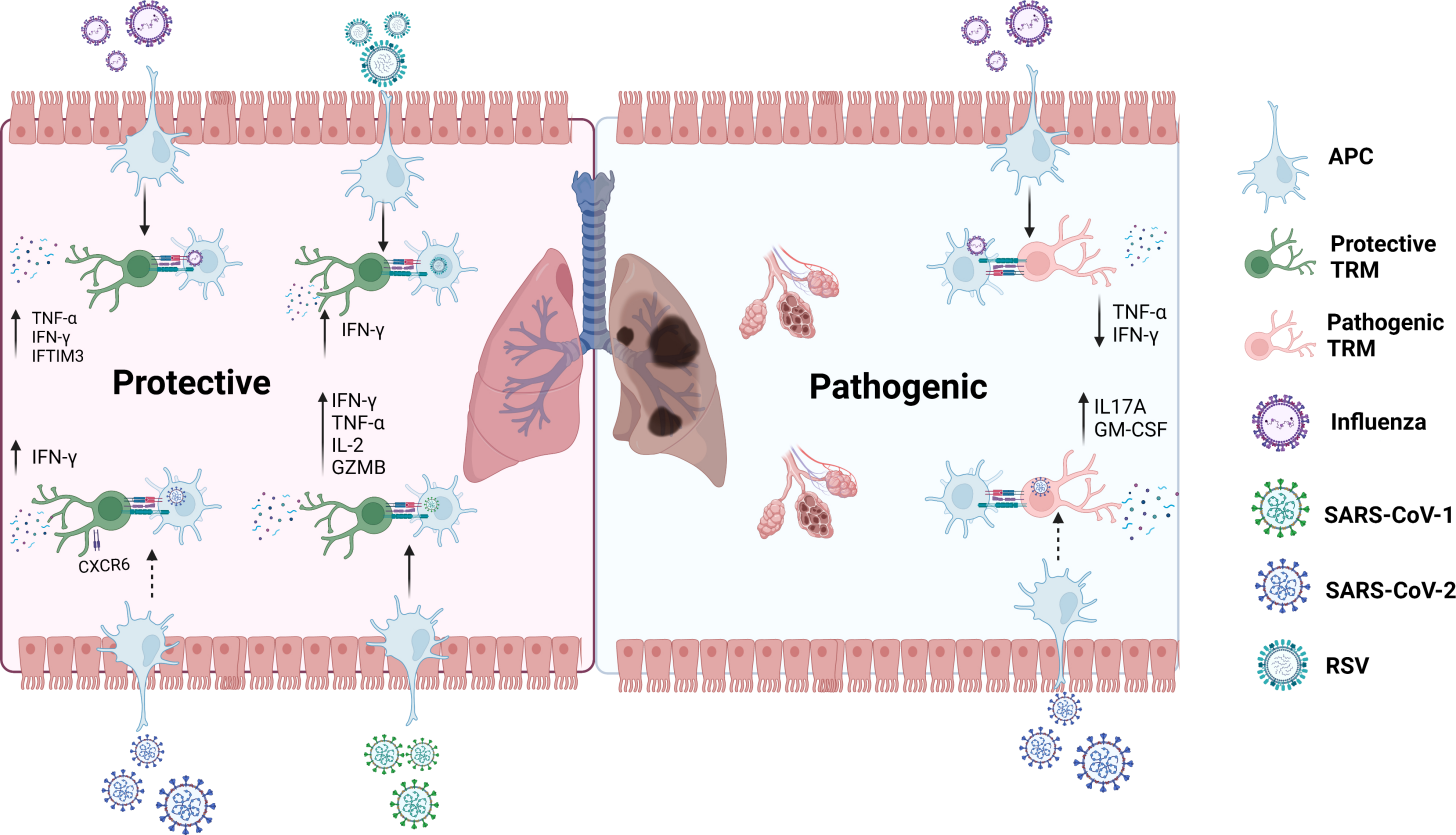
Roles of TRM cells in RSV, Influenza, SARS-CoV-1 and SARS-CoV-2.In the secondary infection, TRM cells rapidly react to protect the host from Influenza, RSV and SARS-CoV-1 through secreting important molecules such as IFN-γ, TNF-α, and IL-2. It may also have the same effect in SARS-CoV-2. On the other side, TRM cells could also arouse pathogenic effects when the host is infected with Influenza, which might also happen in SARS-CoV-2 infection.GZMB: granzyme B. GM-CSF: granulocyte-macrophage colony-stimulating factor. dotted line: possible impact.

In an RSV mouse model, Kinnear *et al.* observed that TRM cells have a protective effect against RSV (13). Compared with mice infected with RSV for the second and third time, mice infected for the first time had significantly lower body weight, increased viral load, and significantly lower serum anti-RSV immunoglobulin after infection. Mice infected multiple times produced numerous antigen-specific CD8^+^ TRM cells in the respiratory tract. Researchers then transferred cells from the respiratory tract of previously infected mice to the respiratory tract of uninfected mice, which partially rescued the weight loss in uninfected mice. Airway CD8^+^ TRM cells decreased weight loss, viral load, and increased IFN-γ, suggesting their protective effect against RSV infection. In RSV infected healthy adult volunteers, pulmonary virus-specific CD8^+^ TRM cells were observed which accumulated extensively during the recovery period (12). During infection, the proportion of CD8^+^ T cells expressing CD103 was upregulated, peaking on day 10 and decreasing in the bronchoalveolar lavage (BAL) as the infection subsided. Enrichment of CD8^+^ TRM cells in the airway was associated with mitigated respiratory symptoms, virus control, and reduced disease severity. This indicates that CD8^+^ TRM cells in human lungs defend against severe respiratory viral disease. RSV-specific CD8^+^ T cells in the BAL of African green monkey were about ten times higher than that in the blood, showing effector memory (CD95^+^CD28^-^)/tissue-resident memory (CD69^+^CD103^+^) T cell phenotype (73). The dynamics of RSV-specific CD8^+^ T cells in blood and BAL were associated with a decrease in the viral titer. Different models have shown that TRM cells protect the host against RSV (**Figure 4**).

### Influenza

Influenza has caused several tragic events in the first half of the twentieth century, killing an estimated 50 million people in the 18 months after the end of World War I (74). It can present diverse conditions, ranging from asymptomatic infections and various respiratory syndromes to fulminant primary viral pneumonia and secondary bacterial pneumonia, damaging all organs. Pulmonary CD8^+^ TRM cells reveal unique traits during influenza infection. CD8^+^ TRM cells are recruited through chemokine (C-X-C motif) receptor 6/chemokine (C-X-C motif) ligand 16 CXCR6/CXCL16 from the lungs to the airway to realize ectopic denfence (17). Interestingly, pulmonary virus-induced CD8^+^ TRM cells can retrogradly migrate from the lungs to medLNs, providing long-term regional memory and recording previous antigen experience to provide protection during secondary virus attack (75).

In the medLNs, accumulation of conventional dendritic cells strengthens T cell initiation during the primary infection and the protective heterosubtypic immunity responses of the CD8^+^ TRM cells (76). Pulmonary TRM cells deposit bronchial-associated lymphoid tissue in the lung parenchyma and provide cross-immune protection against different influenza virus strains (25). Paik et al. (76) demonstrated that mice infected with H3N2 for 3 subsequent weeks could defend against H1N1. These mice displayed decreased virus load and relieved weight loss compared with previously uninfected heterologous mice, suggesting the protective effect of CD8^+^ TRM cells on influenza. Moreover, the use of FTY720 did not impair the heterologous protection, indicating that the protection is independent of the peripheral circulation and may be mediated by secreting TNF-α and IFN-γ (77). CD4^+^ TRM cells also mediate remarkable protection. After influenza infection, CD4^+^ TRM cells isolated from the lungs of mice have been shown to mediate enhanced viral clearance and survival during fatal influenza infections (34, 78).

However, the viability of TRM cells in human requires further investigation. Wu *et al.* (76) found that heterotypic immunity to influenza declined within six to seven months after the initial infection. Nguyende et al. discovered that CD8^+^ TRM cells were the most susceptible to age among all subsets of T cells and age was inversely correlated with CD8^+^ TRM cells in adults (14). This may also explain why older adults are more likely to develop severe clinical symptoms after contracting influenza. In essense, the unstability and waning of CD8^+^ TRM cells is primarly due to apoptosis (79). Nevertheless, the decay may be rescued by repeated antigen exposure. Van *et al.* clarified that repeated exposure to influenza enhanced the persistence of lung CD8^+^ TRM cells and prolonged the durability of heterologous (80).

Despite these factors, some research has introduced new regulatory mechanisms for CD8^+^ TRM cell formation in influenza. In mouse models, TRM cells selectively maintain the interferon-induced transmembrane protein IFITM3, a protein with extensive resistance to viral infection. Expression of IFITM3 in CD8^+^ TRM cells enhances resistance to influenza reinfection and increases CD8^+^ TRM cell maintenance (24). Furthermore, secretion of IFN-γ and restriction of T-bet expression are required for CD8^+^ TRM cell expression, which is accomplished by CD4^+^ T cell (29).

In conclusion, TRM cells can migrate to localized areas and provide long-term regional memory to defend against influenza (**Figure 4**). However, their durability is limited and related to age. Their decline is mainly due to self-apoptosis. Despite IFN-γ secretion by CD4^+^ T cells to upregulate CD8^+^ TRM cells, CD4^+^ TRM cells can reversely contribute to vigorous protection of CD8^+^ T cells (35). Notably, Goplen et al. (81) disclosed their pathogenic role in aged mice, contradicting the previous recognition of CD8^+^ TRM cells. Aged mice infected with H1N1 displayed more influenza-specific TRM cells than young mice, accompanied by extremely weakened heterologous immunity, enhanced pulmonary inflammation, and lung fibrosis. These CD8^+^ TRM cells were unable to secrete IFN-γ and TNF-α. After exhausting CD8^+^ TRM cells in the lungs, the inflammation and fibrosis were relieved. This unexpected discovery hints to more possibile roles of TRM cells in pulmonary Influenza infection.

### SARS-CoV-2

Since the outbreak of COVID-19 in 2019, SARS-CoV-2 specific and vaccine-induced T-cell immune protection has become a popular topic of discussion. A previous study showed that SARS-COV-1-specific CD8^+^ TRM cells can last six years after infection, while memory B cells and viral antibodies cannot be detected in patients recovering from SARS. In addition to reducing viral load, CD8^+^ TRM cells effectively produce a variety of effector cytokines, including IFN-γ, TNF-α, and IL-2, and cytolysate molecules like granzyme B to provide protection (82).

However, we cannot simply regard TRM cells as defence against SARS-CoV-2. SARS-CoV-2-specific CD8^+^ TRM cells were found in the oropharyngeal lymphoid tissue of children and adults unexposed to COVID-19, which were functionally weaker than EBV-specific CD8^+^ TRM cells, possibly spearheading an early immune response. Niessl *et al.* pointed that preexisting CD8^+^ TRM cells may induce heterologous immune responses against COVID-19 (83). The protective role of lung TRM cells against SARS-CoV-2 has also been observed by a three-dimensional perfusion model of human lung tissue (84, 85). Concerning the paired airway and blood samples, Szabo *et al.* observed that in patients previously infected with COVID-19, CD4^+^ and CD8^+^ TRM cells have superiority in the lungs, dominating the airways. Airway T cells expressed upregulation of TRM cell-related gene markers such as CXCR6. More importantly, it upregulated the expression of key cytokines and chemokines, such as IFN-γ, suggesting that TRM cells may protect patients with severe COVID-19 (86). These TRM cells produce IFN-γ in response to *in vitro* stimulation that persist for at least 10 months in the lungs of patients in the covalescent period, highlighting the persistence of TRM cells-related immunity (87). Dai *et al.* exhibited that CD8^+^ TRM cells decreased by 2.4 times in patients with severe infection compared to moderate infection with lower expression of CXCR6, which may protect the lungs in previous experiments (88). Moreover, CD8^+^ TRM cells undergo active expansion in patiens with mild infection, while they perform more naïve funtions in severely infected patients (89). In patients with moderate infection, CD8^+^ TRM cells alleviate inflammation through CXCR6-mediated virus clearance, while the expression of CD8^+^ TRM cells is unstable and reduced in patients with severe infection, leading to viral replication (88) (**Figure 4**). Furthermore, in contrast to influenza, interferon response against SARS-CoV-2 induced by CD8^+^ TRM cells does not decline with age (14) and nasal CD8^+^ TRM cells can last at least two months after virus clearance (31), indicating the persistence of TRM cells against SARS-CoV-2.

However, other studies have challenged the protective effect of CD8^+^ TRM cells during SARS-CoV-2 infection. Roberts et al. (32) elucidated that though lung-resident T cells can be induced by SARS-CoV-2, they cannot provide adequate protection against secondary viral infection. In mice model, transferring T cells from SARS-CoV-2 infected mice to uninfected mice did not improve survival after reinfection. This prompts the unknown efficiency of TRM cells in protecting patients against SARS-CoV-2 during secondary infection. Additionally, many patients in the convalescent stage of COVID-19 infection still experienced respiratory symptoms for months. Vijayakumar *et al.* found that compared to healthy individuals, T cell frequencies in these patients were increased, particularly CD8^+^ TRM cells by immuno-proteomic profiling. The heightened number of CD8^+^ TRM cells was correlated with increased cell death and indicated persistent airway symptoms, namely decreased forced vital capacity (90).

This is evidence that TRM cells predominate during COVID-19 infection. Although some observations of human samples emphasized the possible protective correlation between TRM cells and SARS-CoV-2, studies in mice show that the protection is insufficient. Moreover, exploration of the TRM cells of patients with severe infection and convalescence with respiratory symptoms showed association with ongoing lung injury. Roles of TRM cells in SARS-CoV-2 are complicated. TRM cells at different disease stages and distinct populations may display different effects, being potential pathogenic or protective orchestrators in COVID-19. Furthermore, CD8^+^ TRM cells regulate CD4^+^ T cells to influence immune responses during SARS-CoV-2 infection. Kaneko *et al.* showed that decline of Bcl-6^+^ T follicular helper cells was responsible for the loss of germinal centers and accumulation of activated B cells from non-germinal sources, triggering low efficiency and unsustainable humor immune responses during acute and severe SARS-CoV-2 infection (91). Transcription factor Bcl-6 is indispensable for the development of T follicular helper cells and mutually antagonizes Blimp1 in the process (92, 93). As mentioned above, formation and maintenance of CD8^+^ TRM cells in the lungs requires Blimp1. We assume that by upregulation of Blimp1, CD8^+^ TRM cells inhibit Bcl-6 to impede the generation of T follicular helper cells, affecting humoral immunity in severe COVID-19. More studies are needed to verify this hypothesis and patients with different SARS-CoV-2 infection conditions should also be considered. An update on recognition of long SARS-CoV-2 T cell immunity is needed for more research on the absolute roles of TRM cells in SARS-CoV-2.

## Application in preventive and therapeutic strategies

Till date, there are no drugs that efficiently cure the abovementioned respiratory viruses. Thousands of researchers work on the research and development of treatment. Although B cells eliminate the virus faster in the form of neutralizing antibodies, T cell immunity-related theraputic strategies can maintain long-term protection, and TRM cells largely contribute to immunity *in situ*.

The study and exploration of TRM cells highlights that the site of vaccine injection is critical, and nasal mucosal vaccination may become the trend to curb the replication of respiratory viruses. TRM cells are required for vaccine-induced influenza associated T cell immunity (94). Morabito et al.(95) investigated a murine cytomegalovirus vector vaccine expressing RSV M-protein. Compared with intraperitoneal injection, intranasal inoculation triggered a large amount of CD8^+^ TRM cells, mediating early viral clearance over time. A similar result was observed during influenza vaccine research (96). Mucosal immunity in the respiratory tract also encourages the application of inhaled vaccine. Aerosol inhalation of two doses of Ad5-nCoV, an aerosolised adenovirus type-5 vector-based COVID-19 vaccine, induced production of benign neutralization antibodies with well tolerance and consumed less vaccine dosage than intramuscular injection (97). Inhalation vaccine can both improve compliance to the medication and reduce the cost of vaccination. Enhancement of local mucosal immunity prevents infection and blocks tramsmission at the point of virus invasion, making it safer and more convenient for promotion in population.

TRM cells mediate superior protection in heterotypic infection, highlighting their potential as a universal vaccine target. For production of large amounts of TRM cells, Bosnjak et al. utilized a prime-boost protocol on a novel modified vaccinia virus Ankara (MVA)-SARS-2-spike vaccine candidate to induce immune responses, which alleviated weight loss, increased clinical score, and decreased viral titers in rodents (98). Furthermore, Lei et al. developed a recombinant RBD vaccine against variants of concern for intranasal administration, which not only induced and maintained high IgG levels, but also enhanced mucosal immunity, including lung TRM cells (99). In particular, humoral immunity and TRM cells attained by intranasally delivered SARS-CoV−2 DNA vaccine efficiently suppresses the wild type and Beta variant, providing persistent protection (100). Remarkably, in the absence or with low expression of virus-neutralizing antibody, systemic or pulmonary CD4+ TRM cells and protective CD8 T cells defended effectively against the Beta variant, without lung immunopathology (101). Vaccines targeted at TRM cells are promising for successful prevention of reinfection.

Various combination adjuvants may also improve efficiency by stimulating or maintaining TRM cells. As mentioned above, conventional dendritic cells enhance the protection induced by TRM cells. Wakim et al. developed an antibody targeted inoculation method to present the antigen only to respiratory dendritic cells, facilitating the generation of protective pulmonary TRM cells against influenza (102).

Several TRM cells stimulators, such as IL-1β and zymosan, were used in conjugation with with vaccine, which proved to be more effective in influenza mice models. IL-1β was used as an adjuvant in recombinant adenovirus vector encoding hemagglutinin and nuclear protein in mice. Abundant TRM cells were accumulated and weight loss and virus copies in mice were mitigated. IL-1β and local antigen resulted in activation of key checkpoints in TRM cell formation, including epithelial cell activation, expression of chemokines and adhesion molecules, and recruitment of lung-derived CD103^+^ DCs (103). Without the antigen, only the adjuvant usually cannot drive the generation of TRM cells. Notably, zymosan promoted the differentiation from effector T cells to TRM cells passing by antigen. When used in combination with injectable influenza vaccine, intranasal zymosan significantly increased influenza-specific pulmonary TRM cells (104).

However, there are some complications with treatments based on TRM cells. Firstly, the existence of TRM cells is not stable enough to maintain permanent protection. Using an adenovirus expressing influenza nucleoprotein, Uddback *et al.* demonstrated that CD8^+^ TRM cells in the lungs could sustain for at least a year post vaccination (105). Other than apoptosis, retrocedent migration into medLNs may also explain why TRM cells decrease overtime as described previously, which hinders protection in the lungs. Apparently, the existence of TRM cells is dynamic. Maintenance of protective TRM cells *in situ* is worth exploring. Secondly, the balance between protective and pathogenic roles of TRM cells is unknown. The switching point at which TRM cells change from protective to pathogenic is unknown, especially in the elderly.

TRM cells have supreme value in the treatment of respiratory viruses. In addition to vaccines, new drugs targeted at TRM cells have also been released. Pretreated FC-fused IL-7 protects mice from fatal influenza infection depending on tissue-resident memory-like T cells in the lungs, and lasts for several weeks (106). Utilizing the unique advantages of TRM cells, such as persistence in tissues and remarkable ability to undertake heterotypic immunity, a portable, and highly protective vaccine inducing TRM cells against multiple pathogens can be developed in the future. Moreover, devotion to clinical administration is a big step for vaccines and drugs based on TRM cells.

## Concluding remarks

TRM cells are persistent in non-lymphoid tissues and act as a sentinel in reinfection immunity, responding to infection more quickly. A variety of signaling pathways regulate the generation of TRM cells. However, little is known about the regulation of CD4^+^ TRM cells. Regarding the traits, TRM cells are usually limited *in situ* and migrate localizedly in traditional opinion. As stated above, CD8^+^ TRM cells move to the airway and medLNs to induce effective protection, breaking the stereotype. Nevertheless, TRM cells perform effector-like functions in many tissues and maintain their state without antigen to provide powerful protection (107, 108). Therefore, they are often regarded as terminal differentiation of effector cells, which is actually a semblance. TRM cells expand in the skin and mucosa on contact with the antigen (109, 110), which is quite different from terminally differentiated effector memory (TEMRA) cells. TEMRA cells also reside in situ, providing steady protection during SARS-CoV-2 infection (111), while TRM cells display more plasticity and instability. A recent study showed that reactivational TRM cells in intestine rejoin the lymphoid circulation and have the potential to differentiate into TCM and TEM cells (112, 113). While the phenomenon has not been observed in the lungs, experiments aimed to reconfirm the unstability of TRM cells are still ongoing.

Specifically, TRM cells have different populations. For instance, CD4+ TRM cells have distinct subsets, including Th1, Th2, and Th17 TRM cells (114). Influenza-specific CD4^+^ TRM cells characterize as Th1-like TRM cells secreting IFN-γ and IL-2 and contribute to enhanced protection against influenza (15, 34, 115). Th1 TRM cells provide protection against influenza reinfection and influenza matrix protein ectodomain (M2e)-specific Th17 TRM cells are stimulated through intranasal immunization with M2e adjuvanted with CTA1-DD, generating strong protection against influenza (15, 116). Conversely, in the BAL collected from patients with severe COVID-19, Th17 TRM cells persisted even after virus clearance. These Th17 TRM cells expressing IL-17A and granulocyte-macrophage colony-stimulating factor are potentially pathogenic cytokines and can interact with lung macrophages and cytotoxic CD8^+^ T cells, influencing lung injury (117). Little is known about Th2 TRM cells in respiratory virus infection. Th2 CD4^+^ TRM cells in the peritoneum mediated protective immunity against helminths in mouse intestinal Heligmosomoides polygyrus infection (118). A key research question that needs to be addressed is whether distinct TRM subsets play different roles in reinfection of the same pathogen.

TRM cells exhibit consolidated protection against RSV and influenza, and probable effects on SARS-CoV-2. Consistently, therapeutic strategies targeting TRM cells are good and promising choices to cope with respiratory viruses. This illustrates the bright future of vaccines based on TRM cells in response to respiratory virus. Considering their diverse roles, we may adopt antagonism when TRM cells turn pathogenic. For instance, employing CpG, an agonist of Toll-like receptor, and Il685458, a Notch pathway inhibitor, can ease the airway inflammation caused by formalin-inactivated vaccine, which once failed in prevention of exacerbation of lung disease (119, 120). We believe that insightful application of TRM cells is key to protective mucosal immunity generated by future universal vaccine candidates.

## Author contributions

MZ and ZJ contributed to the central idea and coordinated the writing of the manuscript. NL, YH, TS, and ZJ read, discussed, and revised the manuscript. All authors contributed to the article and approved the submitted version.

## Funding

This study was supported by National Natural Science Foundation of China (NSFC) programs (No. 82070024), Natural Science Foundation of Shanghai (No. 20ZR1443700), Specialized Department Foundation of Minhang District (No: 2020MWTZB02), Plan for leading talent of Minghang District (201807) and Fund from Shanghai Fifth People’s Hospital (2019WYZD03).

## Acknowledgments

The figures in this review were created with Biorender.com.

## Conflict of interest

The authors declare that the research was conducted in the absence of any commercial or financial relationships that could be construed as a potential conflict of interest.

## Publisher’s note

All claims expressed in this article are solely those of the authors and do not necessarily represent those of their affiliated organizations, or those of the publisher, the editors and the reviewers. Any product that may be evaluated in this article, or claim that may be made by its manufacturer, is not guaranteed or endorsed by the publisher.

## Abbreviations

Tissue-resident memory T (TRM) cells; central memory T (TCM) cells; effector memory T (TEM) cells; dendritic cells (DCs); mediastinal lymph nodes (medLNs); interleukin (IL); Krupple-Like Factor 2 (KLF2); sphingosine-1-phosphate receptor (S1PR1); eomesodermin (Eomes); transcription factor 1(TCF1); Respiratory syncytial virus (RSV); bronchoalveolar lavage (BAL).
